# C/EBPα plays a critical role in adipocyte differentiation and obesity

**DOI:** 10.3389/fmolb.2026.1846497

**Published:** 2026-05-20

**Authors:** Xiao Li, Hui Li, Fang Peng, Jianhua Li, Xiaoli Hou, Shaoping Ji

**Affiliations:** 1 Department of Basic Medicine, Zhengzhou Health College, Zhengzhou, Henan, China; 2 Department of Gastroenterology, Huaihe Hospital, Henan University, Kaifeng, Henan, China

**Keywords:** adipocyte differentiation, C/EBPα, lipid metabolism, obesity, transcription regulation

## Abstract

CCAAT/enhancer-binding protein alpha (C/EBPα), a key member of the transcription factor family, inhibits proliferation and plays a central role in adipocyte differentiation, hematopoiesis, and other cellular processes. Its expression and activity are finely regulated at multiple levels, including transcriptional, post-transcriptional, translational, and post-translational modifications. During adipocyte differentiation, C/EBPα participates in establishing a complex regulatory network by coordinating with other transcription factors and modulating downstream key genes such as peroxisome proliferator-activated receptor γ (PPARγ). In obesity, dysregulation of C/EBPα is closely associated with impaired adipocyte differentiation and abnormal lipid metabolism, often contributing to lipid accumulation and hyperlipidemia. As research advances, therapeutic strategies targeting C/EBPα have opened new avenues for obesity intervention. However, numerous challenges remain in translating basic research into clinical applications. This review aims to elucidate the mechanisms and research advances regarding the role of C/EBPα in adipocyte differentiation and obesity.

## 1 Introduction

Adipocyte differentiation is a critical process for energy storage and metabolic homeostasis, involving a highly coordinated and multistep program in which mesenchymal stem cells (MSCs) commit to the adipogenic lineage, differentiate into preadipocytes, and ultimately develop into lipid-laden mature adipocytes (Gregoire et al., 1998). Under normal physiological conditions, adipocyte differentiation, hypertrophy, and programmed cell death (apoptosis) collectively maintain adipose tissue turnover and homeostasis. This dynamic balance is essential for preserving adipose tissue architecture and functional capacity, thereby contributing to the regulation of systemic energy balance, insulin sensitivity, lipid metabolism, and inflammatory signaling (Ali et al., 2013; Huh and Romero, 2022).

However, with the rapid development of the global economy, significant lifestyle changes, and increasingly unhealthy dietary patterns, the prevalence of obesity is rising at an alarming rate and has become a major public health threat worldwide (Stefan et al., 2013; Preda et al., 2023). Obesity is not merely an increase in body weight; at its core, it results from excessive adipocyte proliferation, abnormal differentiation, and dysregulated lipid metabolism, leading to pathological expansion of adipose tissue (Hagberg and Spalding, 2024; Ambele et al., 2020). At the cellular level, obesity is associated with an increase in adipose tissue mass, which depends on both the size and number of adipocytes (Ying and Simmons, 2020). Adipogenesis is the process by which preadipocytes differentiate into mature fat cells (Wang et al., 2020). Therefore, the process of adipocyte differentiation and its regulatory mechanism have become important issues in obesity research (Gorelick, 2022). Excess adipose tissue, particularly visceral adipose tissue, triggers a cascade of metabolic disorders, including insulin resistance, type 2 diabetes mellitus, dyslipidemia, hypertension, and cardiovascular diseases, severely compromising patients’ quality of life and lifespan (Wang et al., 2022; Taylor, 2021; Stefan, 2020). Therefore, elucidating the molecular mechanisms underlying adipocyte differentiation and identifying key regulatory targets for abnormal adipogenesis hold critical theoretical and practical significance for developing prevention and treatment strategies against obesity and related metabolic disorders.

The molecular mechanisms related to adipogenesis have been widely studied. CCAAT region/enhancer binding protein (C/EBP) has the characteristic of heat resistance and can bind to DNA through the alkaline region, relying on the leucine zipper structure to form homologous or heterodimers to exert its function (Ramji and Foka, 2002). The C/EBP family consists of C/EBP-α, β, δ, γ and ε. This protein family exhibits bidirectional regulatory characteristics. It can not only positively activate the transcription of target genes but also negatively inhibit the expression of specific genes, participating in normal metabolic regulation and the occurrence and development of diseases (Tsukada et al., 2011). C/EBPα as a core transcription factor regulating adipocyte differentiation, is indispensable for the initiation, maintenance, and functional maturation of differentiation (Lee et al., 2019). Its expression and activity are modulated by multiple mechanisms, including transcriptional regulation, post-translational modifications, and protein interactions, in turn, its abnormalities in these processes can disrupt normal adipocyte differentiation (Chowdhury et al., 2023). C/EBPα regulates a cascade of downstream transcription factors involved in adipocyte differentiation, forming a hierarchical regulatory network. Meanwhile, adipocyte differentiation is coordinately controlled by multiple factors, and other transcription factors interact intricately with C/EBPα, collectively constituting a sophisticated regulatory network (Josan et al., 2021).

Under obese conditions, dysregulated expression and function of C/EBPα are closely associated with lipid metabolism abnormalities and hyperlipidemia, leading to impaired adipocyte differentiation and disturbances in lipid metabolic homeostasis, thereby exacerbating obesity and metabolic disorders (Kim et al., 2025).

We will highlight that C/EBPα, as a core transcription factor in adipocyte differentiation, has been extensively studied across multiple regulatory levels, revealing not only the key molecular basis of adipogenesis and energy metabolism but also providing critical insights into the pathogenesis of obesity and related metabolic disorders. Dysregulation of the C/EBPα regulatory network is a central event in abnormal adipose tissue expansion and lipid metabolic disturbances. Therefore, therapeutic strategies targeting C/EBPα and/or its modulator(s) hold a promise for opening new avenues in obesity treatment. By precisely modulating its expression and activity, such approaches can facilitate the translation of basic research into clinical applications, offering theoretical support and new directions for the management of metabolic diseases.

## 2 Gene structure and characteristics of C/EBPα

The gene encoding C/EBPα exhibits high conservation across different species, reflecting its crucial biological functions in the evolutionary processes. Taking the *CEBPA* and *Cebpa* genes as examples, they demonstrate significant similarities in gene sequence and structure (Ramji and Foka, 2002). The human *CEBPA* gene is located on the chromosomal region 19q13.1, with a cDNA length of approximately 2,385 base pairs and contains multiple translation initiation sites. This gene consists of multiple exons and introns: exons encode the amino acid sequence of the protein, while introns play important roles in processes such as gene expression regulation (Lekstrom-Himes and Xanthopoulos, 1998). The mouse *Cebpa* gene also exhibits a similar structural composition, with an extremely high sequence similarity to the human gene in key functional regions (Antonson and Xanthopoulos, 1995). Emerging evidence indicates that the *Cebpa* gene, although intronless, generates multiple protein isoforms---notably p42 and p30---through alternative translation initiation (Lin et al., 1993; Fernandez et al., 2024). The p30 isoform, lacking the N-terminal domain responsible for antiproliferative activity, plays a distinct role in maintaining an undifferentiated state, potentially contributing to the fine-tuned regulation of adipocyte differentiation (Lin et al., 1993). To date, no non-coding pseudogenes of *Cebpa* have been definitively identified. However, several long non-coding RNAs, such as ADINR and MSTRG.12568.2, have been shown to regulate *Cebpa* expression (Xiao et al., 2021; Lin et al., 2022). Future studies should systematically investigate the existence of potential C/EBPα pseudogenes and elucidate the specific roles of its isoforms in adipocyte differentiation and metabolic regulation.

The protein encoded by the *Cebpa* gene belongs to the basic leucine zipper (bZIP) transcription factor family, characterized by a typical bZIP structural domain. This domain consists of a DNA-binding region rich in basic amino acids and a leucine zipper region (Landschulz et al., 1988). The leucine zipper facilitates the formation of dimeric structures, enhancing C/EBPα′s DNA-binding ability (Landschulz et al., 1989). This enables it to specifically recognize and bind to the promoter or enhancer regions of target genes, thereby regulating the transcriptional process (Miller et al., 2003). The C/EBPα protein also contains other functional domains, such as transcriptional activation domains and transcriptional repression domains. These domains interact with other transcription factors or co-regulators to cooperatively modulate gene expression (Friedman, 2015).

Studies have shown that the conserved structure and characteristics of the *Cebpa* gene play similar critical roles in adipocyte differentiation across different species. In mouse models of adipocyte differentiation, knockout of the *Cebpa* gene leads to impaired adipocyte differentiation, preventing the formation of mature adipocytes. This indicates that the *Cebpa* gene is essential for the normal differentiation of adipocytes. Similarly, research on human adipocytes has demonstrated that the expression level and activity of the *CEBPA* gene are closely associated with the degree of adipocyte differentiation. Abnormal expression or functional loss of C/EBPα can result in aberrant adipocyte differentiation, thereby affecting lipid metabolism and energy balance (Rosen et al., 2002).

As a transcription factor, the function of C/EBPα depends on its interaction partners, which remain largely unknown. Further research into its isoforms and upstream regulation is needed to reveal its core role in adipocyte differentiation and metabolism.

## 3 Regulation of C/EBPα expression and activity

C/EBPα, a key member of the C/EBP family of transcription factors, plays a critical role in adipocyte differentiation and is also involved in cell differentiation, metabolic regulation, and inflammatory responses (Lekstrom-Himes, 2001). The expression and activity of C/EBPα are regulated by multiple mechanisms, including transcriptional regulation, post-translational modifications (PTMs), protein-protein interactions, and microRNA (miRNA) -mediated regulation.

Multiple up-stream transcription factors are involved in the transcriptional regulation of the *Cebpa* gene expression. AP-1 (Activator Protein-1), as an important transcription factor, can bind to specific sequences within the promoter region of the *Cebpa* gene. AP-1 is composed of proteins such as c-Jun and c-Fos, which function by forming heterodimers (Hong et al., 2011). When cells are stimulated by external signals such as growth factors or cytokines, AP-1 becomes activated and binds to the *Cebpa* gene promoter, thereby promoting the transcription of the *Cebpa* gene. Studies have shown that during the early stages of adipocyte differentiation, stimulation by growth factors leads to the activation of AP-1, which subsequently upregulates the expression of the *Cebpa* gene and drives the adipocyte differentiation process (Farmer, 2006; Rosen and MacDougald, 2006).

Although the synergistic role of AP-1 in initiating C/EBPα expression during adipogenesis is well established, the specific composition of its interacting partners may vary depending on the cellular context and developmental stage. It can be speculated that under different physiological or pathological conditions—such as inflammation, tissue regeneration, and metabolic stress—AP-1 may regulate *Cebpa* transcription by associating with distinct co-regulators, chromatin remodeling complexes, and other factors. The transcription factor SP1 (Specificity Protein 1) also plays a significant role in the transcriptional regulation of the *Cebpa* gene. SP1 is rich in GC-box-binding domains and can specifically recognize and bind to GC-box sequences in the promoter region of the *Cebpa* gene (Zeng et al., 2020). Through this binding, SP1 can recruit other transcriptional co-factors to form the transcription initiation complex, enhancing the binding of RNA polymerase to the promoter and thereby promoting the transcription of the *Cebpa* gene. During adipocyte differentiation, changes in the expression level and activity of SP1 are closely related to the transcription of the *Cebpa* gene. Knocking down the expression of SP1 significantly inhibits the transcription of the *Cebpa* gene, which in turn affects adipocyte differentiation (Tang et al., 1999). Both AP-1 and SP1 promote C/EBPα transcription and regulate adipocyte differentiation. AP-1 is primarily activated by extracellular signals, responding to stimuli such as growth factors to initiate gene expression in an inducible manner. In contrast, SP1 contains GC-rich binding domains, recruits the transcriptional complex, and maintains stable basal transcription levels. The synergistic action of these two transcription factors not only sustains normal lipid metabolism but may also serve as potential intervention targets in metabolic diseases such as inflammation and obesity.

Prior to the initiation of differentiation, a nuclear protein known as C/EBPα undifferentiated protein (CUP) is present in preadipocytes. This protein binds to specific regions of the *Cebpa* promoter, forming a repressive complex that maintains the *Cebpa* gene in a silenced state. As differentiation begins, the activity of CUP gradually diminishes, lifting the inhibition on C/EBPα. This allows the autoregulatory loop to initiate robust expression of C/EBPα (Jiang et al., 1998). In addition, epigenetic regulation also contributes to the control of C/EBPα expression. The histone demethylase plant homeodomain finger protein 2 (PHF2) interacts with C/EBPα and promotes its transcriptional activation by removing repressive histone H3K9 methylation marks. This process enhances the efficiency of adipocyte differentiation (Lee et al., 2014; Okuno et al., 2013).

At the protein level, the activity of C/EBPα is also regulated by various mechanisms, including protein stability, and subcellular localization. Regarding protein stability, in addition to the classical E3 ubiquitin ligase F-box and WD repeat domain-containing protein 7 (*Fbxw7*), recent studies have identified that Atrophin-1 interacting protein 4 (AIP4) promotes the K48-linked ubiquitination of C/EBPα, leading to its proteasomal degradation and thereby negatively regulating adipocyte differentiation (Chowdhury et al., 2023). Overall, current research on the regulation of C/EBPα protein levels remains relatively fragmented. Kim et al. highlighted the critical role of post-translational modifications in regulating C/EBPα function, yet significant gaps still exist in this field (Kim et al., 2024). It is likely that additional molecules regulating C/EBPα expression remain to be identified, particularly across diverse cellular contexts and processes.

## 4 Downstream targets of C/EBPα and adipogenesis-related genes

Transcription factors (TFs) are a specialized group of proteins, including nuclear receptors, that regulate transcription and thereby control gene expression and protein synthesis. Among them, PPARγ, C/EBPα, and sterol regulatory element-binding protein 1c (SREBP-1c) play crucial roles in adipogenesis, lipid and cholesterol homeostasis (Lee and Young, 2000; Lemon and Tjian, 2000). C/EBPα is a core regulatory factor in adipocyte differentiation. It directly binds to C/EBP response elements (CREs) located in the promoter or enhancer regions of downstream target genes, thereby regulating adipogenesis through transcriptional activation or repression. Its regulation of downstream transcription factors represents a critical mechanism through which it drives adipocyte differentiation (Boughanem et al., 2019). The function of C/EBPα spans the entire process of mature adipocyte formation, from inhibiting preadipocyte proliferation and cell cycle exit to lipid droplet formation.

Adipocyte differentiation is a highly orchestrated process governed by a transcription factor cascade among which PPARγ and C/EBP family members are the major regulators (Ghaben and Scherer, 2019; Lefterova et al., 2014). Among the downstream targets related to adipogenesis, PPARγ is the most core cofactor. In the early stage of adipocyte differentiation, C/EBPα can directly bind to the promoter region of the PPARγ gene, promoting its transcriptional activation. The ectopic expression of PPARγ or C/EBPα in fibroblasts can induce lipid conversion (Freytag et al., 1994). In the early stage of differentiation, C/EBPβ and C/EBPδ are rapidly induced to express. As early response co-factors, they can activate the expression of C/EBPα and PPARγ, thereby initiating the entire differentiation process (Tang and Lane, 2012). These factors then work cooperatively to induce the expression of genes responsible for the mature adipocyte phenotype (Tontonoz and Spiegelman, 2008).

During the mid-phase of adipocyte differentiation, C/EBPα also regulates the activity of SREBP-1c, a key transcription factor involved in lipid synthesis (Rai et al., 2021). Specifically, C/EBPα not only directly binds to the CRE in the promoter region of the SREBP-1c gene to enhance its transcription but also indirectly upregulates SREBP-1c expression through activation of PPARγ.

In addition, C/EBPα directly regulates the expression of a series of key genes for fat formation. SREBP-1c, also known as adipocyte determination and differentiation-dependent factor 1 (ADD1), is regulated by C/EBP transcription factors (Eberlé et al., 2004). Furthermore, SREBP-1c contributes to the expression of PPARγ and the production of endogenous ligands for PPARγ (Kim et al., 1998).

The expression and activity of C/EBPα are subject to multi-layered regulation and play critical roles in adipogenesis, metabolism, and inflammation. However, its entire regulatory network remains to be understood: the molecular identity of CUP factors is unknown, the dynamic coordination between AP-1 and SP1 during early differentiation remains unclear, and the AIP4-mediated degradation mechanism lacks *in vivo* evidence. Future research should integrate existing mechanisms with *in vivo* validation to provide more reliable targets for metabolic disease intervention.

## 5 Transcription factors regulating adipocyte differentiation

**FIGURE 1:**
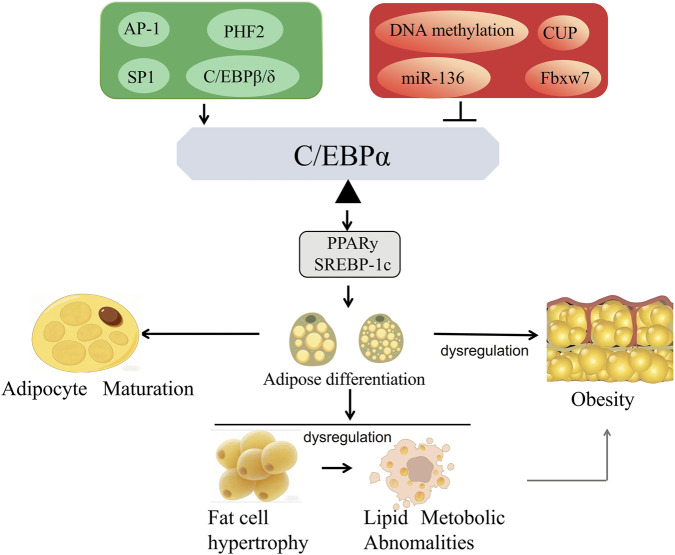
Regulatory mechanism of C/EBPα in adipocyte differentiation. The expression of C/EBPα is positively regulated by AP-1, SP1, and C/EBPβ/δ, and negatively regulated by CUP and miR136. A balance is essential in physical status. Activated C/EBPα subsequently activates downstream targets such as PPARγ and SREBP-1c, thereby regulating the differentiation of white, brown, and beige adipocytes. Disruption of adipogenic homeostasis can lead to various physiological and pathological outcomes, including adipocyte maturation, obesity, and abnormal lipid metabolism even diabetes. Abbreviations: AP-1, activator protein-1; SP1, specificity protein 1; CUP, C/EBPα undifferentiated protein; miR136, microRNA 136; PPARγ, peroxisome proliferator-activated receptor γ; SREBP-1c, sterol regulatory element-binding protein 1c.

Adipocyte differentiation is a critical process in the regulation of systemic energy homeostasis, involving a complex cascade from the commitment of mesenchymal stem cells to preadipocytes and subsequent terminal differentiation into mature adipocytes. The precise regulation of transcription factors serves as the central driving force behind this orderly progression (Wang et al., 2024). As a key nodal molecule in adipogenesis, C/EBPα is not only essential for the terminal differentiation of adipocytes but also a core master regulator in maintaining the phenotype of mature adipocytes. Elucidating its regulatory mechanisms holds significant importance for understanding the pathogenesis of obesity and metabolic syndromes (Figure 1).

In the process of white adipocyte differentiation, C/EBPα occupies a core downstream position in the adipogenic transcriptional cascade. In the classical 3T3-L1 preadipocyte differentiation model, hormonal induction first triggers the expression of early transcription factors C/EBPβ and C/EBPδ (May et al., 2001). These factors activate downstream target genes, leading to growth arrest and preparing the cells for terminal differentiation. Subsequently, the expression of C/EBPα is markedly upregulated (Mandrup et al., 1998).

Experimental studies have confirmed that inhibiting *Cebpa* expression via antisense RNA completely blocks the adipogenic program in 3T3-L1 cells. This not only prevents the activation of adipocyte-specific genes but also inhibits the accumulation of cytoplasmic triglycerides (Liu et al., 2018). Conversely, introducing a sense *Cebpa* expression vector can restore the differentiation phenotype and even trigger the differentiation process without exogenous hormonal induction. These findings demonstrate that C/EBPα is both a necessary and sufficient factor for adipocyte differentiation (Jin et al., 2000).

During the initiation phase of adipocyte differentiation, the transcription factors C/EBPβ and C/EBPδ are rapidly induced as early-response regulators. Functioning as upstream master switches, they initiate the entire differentiation program by activating the expression of downstream core transcription factors C/EBPα and PPARγ. These factors act synergistically to drive the gene expression profile characteristic of the mature adipocyte phenotype (Lee et al., 2020). The study found that in 3T3-L1 cells, depletion of Ajuba significantly reduced both mRNA and protein levels of PPARγ and C/EBPα and impaired white adipocyte differentiation, while overexpression increased the expression of these genes and promoted white adipocyte differentiation. Moreover, restoring the expression of either C/EBPα or PPARγ in Ajuba-deficient 3T3-L1 cells ameliorated the impaired lipid accumulation (Yan et al., 2022).

MiRNAs can greatly influence many biological processes of obesity, such as adipogenesis, lipid metabolism and homeostasis (Elkhawaga et al., 2023). The miR136 simulation experiment to block lipid droplet formation indicated that miR136 inhibited the white adipocyte differentiation of preadipocytes and further reduced the expression of PPARγ and *Cebpa* (Luo et al., 2023).

C/EBPα is a core regulator of adipocyte differentiation, governing the process through positive feedback and synergy with PPARγ. Current studies on its upstream regulatory mechanisms are mostly based on *in vitro* models and lack *in vivo* dynamic validation. Future efforts should integrate single-cell multi-omics approaches to elucidate its regulatory mechanisms under physiological and pathological conditions (Table 1).

**TABLE 1:** C/EBPα function in white vs. brown/beige adipogenesis.

Comparison Dimension	White adipocyte differentiation	Brown/Beige adipocyte differentiation
Differentiation Orientation	Energy storage, forming mature lipid-storing cells with a single large lipid droplet.	Energy expenditure, forming thermogenic cells with multiple small lipid droplets and high mitochondrial content; beige adipocytes can be derived from the browning of white adipocytes
Core localization of C/EBPα	Acts as a core transcription factor, cooperates with PPARγ to activate downstream lipid synthesis-related genes, and promotes lipid droplet accumulation and mature adipocyte formation	Also induced to express, but participates in a different differentiation network and is more involved in the regulation of thermogenic genes
Upstream activators	C/EBPβ, C/EBPδ, AP-1, SP1, PHF2	C/EBPβ, C/EBPδ
Core downstream target genes	PPARγ, SREBP-1c	PPARγ + thermogenic-specific genes
Negative regulators	miR136, CUP protein, AIP4, *Fbxw7*, obesity-related inflammation/DNA methylation	No specific negative regulators, shares some universal repressors with white adipocytes
Specific regulatory Pathways/Substances	Ajuba: Indirectly promotes C/EBPα expression; Cav-2: Drives the nuclear localization of C/EBPα and promotes adipocyte hypertrophy	atRA: Inhibits C/EBPα and induces browning of white adipocytes; XIST-PPARγ-C/EBPα axis: thermogenic directional regulation
Pathological association	Excessive activation leads to hyperplasia and hypertrophy of white adipocytes, promoting obesity	Activation helps increase energy expenditure and counteract obesity

Abbreviations: SREBP-1c, sterol regulatory element-binding protein 1c; AP-1, activator protein-1; SP1, specificity protein 1; PHF2, plant homeodomain finger protein 2; miR136, microRNA, 136; CUP, C/EBPα, undifferentiated protein (a nuclear repressor); AIP4, atrophin-1, interacting protein 4; *Fbxw7*, F-box and WD, repeat domain-containing protein 7 (mouse gene); Ajuba, a LIM, domain protein; Cav-2, caveolin-2; atRA, all-trans retinoic acid; XIST, X-inactive specific transcript.

White and brown/beige adipocyte differentiation play distinct yet complementary roles in obesity and diabesity. White adipocytes primarily store excess energy as triglycerides, and their expansion is closely associated with insulin resistance, chronic inflammation, and metabolic dysfunction. In contrast, brown and beige adipocytes dissipate energy through thermogenesis, enhancing energy expenditure and improving glucose and lipid metabolism. Promoting brown/beige adipocyte differentiation has emerged as a promising therapeutic strategy to counteract obesity by increasing caloric burning and insulin sensitivity. Therefore, while white adipocyte differentiation contributes to disease progression, brown/beige adipocyte differentiation offers protective metabolic effects, highlighting the importance of balancing these processes in managing obesity and related metabolic disorders. Understanding the distinct regulatory roles of C/EBPα in white versus brown/beige adipocyte differentiation may provide valuable insights for clinical practice and translational medicine.

## 6 Dysregulation of C/EBPα in obesity

C/EBPα plays a critical role in regulating lipid metabolism and energy balance as well. It participates in the transcriptional control of genes related to fatty acid uptake, triglyceride synthesis, and lipolysis (Jakab et al., 2021; Harasymiak-Krzyżanowska et al., 2013).

Under conditions of obesity, both animal models and human studies have revealed significant alterations in the expression and activity of C/EBPα. In high-fat diet-induced obese mouse models, the mRNA levels of *Cebpa* and protein levels of C/EBPα in adipose tissue are markedly elevated. Studies have shown that after 8 weeks of long-term high-fat diet feeding, mice exhibited significant weight gain, and the expression of *Cebpa* in adipose tissue increased approximately 2–3 fold compared to the normal diet group (Le Bacquer et al., 2007). This upregulation in expression may result from increased lipid accumulation within adipocytes induced by the high-fat diet, leading to alterations in the intracellular environment and subsequent activation of a series of signaling pathways that promote the transcription and expression of the *Cebpa* gene.

In adipose tissue samples from obese individuals, similar abnormal changes in *CEBPA* expression have been observed. A study comparing obese patients with normal-weight controls found that the protein expression levels of C/EBPα were significantly higher in both subcutaneous and visceral adipose tissues of obese individuals compared to those of normal-weight subjects, and these levels positively correlated with body mass index (BMI) (Jannat Ali Pour et al., 2023). This suggests that the upregulation of C/EBPα expression may be an important feature during the development and progression of obesity, and its elevated expression is likely closely associated with both adipose tissue expansion and functional abnormalities in obese patients.

Currently, no study has directly reported an upregulation of C/EBPα transcriptional activity under obese conditions. However, based on its regulatory mechanisms, it can be speculated that the activation of upstream signaling pathways in obesity may enhance C/EBPα activity through phosphorylation, as studies have shown that phosphorylation at Ser-21 is critical for its transcriptional function. Additionally, changes in the expression of co-activators may also modulate its transcriptional activity (Cha et al., 2008). Therefore, the enhanced function of C/EBPα in obesity may not only result from increased expression but also involve alterations in its activity at the level of post-translational modifications (Kim et al., 2024).

Wu et al. demonstrated that C/EBPα and PPARγ cross-regulate each other, governing the transcriptional pathways involved in adipogenesis and insulin sensitivity. This interaction is essential for maintaining lipid homeostasis in adipocytes. The study using the 3T3-L1 preadipocyte model revealed that yeast vacuoles significantly inhibit the differentiation process of 3T3-L1 cells. The mechanism involves the lipases present in the vacuoles interfering with the expression maintenance of key early differentiation transcription factors C/EBPβ and C/EBPδ, thereby obstructing the transcriptional activation of master regulators C/EBPα and PPARγ during the mid-to-late stages of differentiation. This cascade effect ultimately suppresses the differentiation of preadipocytes into adipocytes (Choi et al., 2023a). Studies have shown that obesity-related inflammation can downregulate *CEBPA* mRNA levels in human preadipocytes. Additionally, abnormal DNA methylation in the *CEBPA* promoter region further suppresses its expression, exacerbating differentiation impairment (Couturier et al., 2012; McAllan et al., 2023). Dysfunctional adipocytes are characterized by dysregulated lipid metabolism and decreased insulin sensitivity. C/EBPα regulates the expression of genes involved in lipid metabolism, and its dysfunction can lead to abnormal lipid accumulation. At the same time, it directly participates in the regulation of the insulin signaling pathway. Its deficiency impairs the activation efficiency of insulin receptors, reduces glucose uptake capacity, and exacerbates insulin resistance. Animal experiments have confirmed that *Cebpa* deficiency in adipose tissue can induce hepatic steatosis and promote the development of metabolic syndromes such as type 2 diabetes (Inoue et al., 2004).

Caveolin-2 (Cav-2), as a key molecule controlling adipocyte hypertrophy, during the hypertrophy stage of adipogenesis, the binding of phosphorylated Cav-2 to lamellar protein A/C promotes the separation of C/EBPα and PPARγ from lamellar protein A/C, thereby promoting adipocyte hypertrophy induced by C/EBPα and PPARγ (Choi et al., 2023b). Furthermore, the ubiquitin ligase *Fbxw7* regulates the stability of C/EBPα by targeting it for degradation. During obesity, abnormal expression of *Fbxw7* accelerates the degradation of C/EBPα, amplifying its dysfunctional effects (Bengoechea-Alonso and Ericsson, 2010).

In summary, the aberrant expression and dysfunction of C/EBPα serve as a critical link connecting obesity, adipocyte dysfunction, and insulin resistance, with such abnormalities primarily concentrated in the white adipocyte differentiation pathway mediated by C/EBPα. The overexpression and functional dysregulation of C/EBPα in white adipose tissue drive hyperplasia, hypertrophy, and lipid metabolic disorders in white adipose tissue, representing the core molecular mechanism underlying the development of obesity. In contrast, the regulatory role of C/EBPα in brown/beige adipocyte differentiation remains unaffected under obese conditions, and its insufficient activation instead impairs the body’s energy expenditure capacity, further promoting obesity progression. This differential regulatory characteristic of C/EBPα in the two adipocyte differentiation pathways provides a theoretical basis for targeted interventions in metabolic syndrome. A therapeutic strategy that selectively inhibits the C/EBPα regulatory network in white adipose tissue while activating its regulatory function in brown/beige adipose tissue may serve as an effective approach for obesity treatment (Figure 1).

## 7 Targeting C/EBPα therapeutic strategies and future directions

C/EBPα, as the core transcription factor for adipocyte differentiation, plays a crucial role in fat generation, lipid metabolism, and energy homeostasis regulation. Its abnormal activation is an important molecular mechanism for obesity and related metabolic disorders. Targeting C/EBPα to intervene in the differentiation and metabolic processes of adipocytes has become an important direction for developing obesity treatment strategies. Relevant studies have confirmed the feasibility and potential of various intervention strategies.

The intervention strategy of targeting C/EBPα with natural compounds offers safety advantages and has become a research hotspot. Epigallocatechin gallate can downregulate *Cebpa* expression by inhibiting the ERK pathway, thereby blocking adipogenesis (Kim and Sakamoto, 2012). Caffeine and chlorogenic acid from coffee, when delivered via solid lipid nanoparticles, can significantly inhibit adipocyte differentiation by targeting the PPARγ/C/EBPα pathway, achieving an efficacy improvement of 45.8% compared to conventional extracts. The nano-delivery system effectively addresses the issue of low bioavailability of natural compounds (Uner and Macit Celebi, 2023). Furthermore, the vitamin A derivative all-trans retinoic acid (atRA) can inhibit *Cebpa* expression through the CRABP-II/RARγ axis, while also inducing white adipose tissue browning and increasing energy expenditure (Bilikozen Aygun et al., 2026).

In recent years, synthetic compounds have achieved significant progress in the field of targeted regulation of adipogenesis via C/EBPα, by leveraging structural optimization to achieve specific pathway intervention while overcoming the inherent limitation of low bioavailability associated with natural products (Inthanon et al., 2025). In terms of directly targeting C/EBPα, a synthetic derivative derived from Salvia miltiorrhiza specifically inhibits *Cebpa* expression by activating the ATF3 pathway, leading to significant reductions in body weight and triglyceride levels in a high-fat diet mouse model (Wu et al., 2022a). Similarly, the synthetic TAT38 polypeptide potently blocks 3T3-L1 cell differentiation by downregulating adipogenic factors such as C/EBPα and PPARγ (Park et al., 2024).

In the area of novel target development, Zhou et al. identified the leucine rich repeat (in Flightless I) interacting protein 1 (LRRFIP1)/E2F transcription factor 6 (E2F6)/C/EBPα transcriptional axis, demonstrating that modulating LRRFIP1 expression significantly affects white adipocyte differentiation, thereby providing a new target for drug development (Zhou et al., 2025). Additionally, 1-methyl-1,2,3,4-tetrahydro-β-carboline-3-carboxylic acid (MTCA), identified from yeast hydrolysate, inhibits lipid accumulation by downregulating SREBP and C/EBPα-related pathways (Kim et al., 2023). Synthetic compounds, through structural optimization and the exploration of novel targets, offer a wealth of candidate molecules for anti-obesity drug development, presenting promising prospects for clinical application. Molecular regulators and microbial agents offer diverse strategies for targeting C/EBPα. The long non-coding RNA X-inactive specific transcript (XIST) can directly bind to C/EBPα to promote brown adipocyte differentiation. In obese mice induced by a high-fat diet, overexpression of XIST significantly ameliorated metabolic disorders, providing a novel approach for RNA-based targeted therapy (Wu et al., 2022b).

Among microorganism-derived bioactive substances, heat-killed *Enterococcus faecalis* EF-2001 has been shown to reduce lipid accumulation by inhibiting the expression of *Cebpa* and PPARγ in the insulin signaling pathway. Oral administration of EF-2001 decreases body weight and lowers serum cholesterol and triglyceride levels in high-fat diet-induced obese rats (Lee et al., 2022). Additionally, chaperonin 60 (Cpn60) from Propionibacterium freudenreichii MJ2 suppresses lipid accumulation in 3T3-L1 adipocytes by upregulating Gata2/3 expression and inhibiting the nuclear translocation of C/EBPβ, thereby downregulating *Cebpa* and PPARγ expression and exerting anti-adipogenic effects (An and Lim, 2023). Nano carriers loaded with active substances further enhance targeting efficacy. In addition to gold nanoparticles loaded with plant extracts, which activate the AMPK pathway to downregulate adipogenic genes such as *Cebpa*, thereby achieving dual effects of fat reduction and improved energy metabolism, conjugated linoleic acid (CLA)-loaded tocopherol nanostructured lipid carriers enhance the delivery efficiency of CLA to adipocytes. This approach inhibits fat accumulation and ameliorates metabolic disorders in obese rats by downregulating the expression of *Cebpa* and lipid metabolism-related enzymes (Hsu et al., 2024).

These studies improve metabolic disorders by directly or indirectly regulating C/EBPα and its downstream pathways. Their effectiveness confirms the central role of C/EBPα in adipogenesis and metabolic regulation. Only when this target and its downstream axis are sufficiently critical can interventions achieve significant anti-obesity and lipid-lowering effects. In terms of gene regulation tools, the catalytically dead Cas9 fused to the Krüppel-associated box (dCas9-KRAB) system can epigenetically inhibit the enhancer activity of the *Cebpa* gene, significantly downregulating *Cebpa* expression. This effectively suppresses 3T3-L1 adipocyte differentiation and adipose tissue development in mice, providing a novel tool for precise targeted intervention (Li et al., 2024). Therapies targeting C/EBPα must balance specificity with systemic safety to avoid off-target effects.

Current research mainly focuses on inhibiting its expression or downstream binding, while future efforts should develop specific inhibitors and optimize targeted delivery. Given the extensive crosstalk between C/EBPα and PPARγ, insulin signaling, and inflammatory networks, multi-target or combination strategies hold promise for enhancing efficacy and reducing compensatory mechanisms, offering improved therapeutic prospects for obesity and related metabolic disorders.

## 8 Summary and future perspectives

As the core transcription factor regulating adipocyte differentiation, C/EBPα specifically recognizes target gene promoters through its highly conserved bZIP domain. Under the multi-level regulation of transcription factors, epigenetic modifications, and miRNAs, it forms a synergistic network with PPARγ to co-activate downstream lipid metabolism genes such as SREBP-1c, thereby driving the differentiation of preadipocytes into mature adipocytes and maintaining lipid homeostasis. Under obese conditions, the expression and function of C/EBPα become dysregulated, leading to excessive adipocyte proliferation, lipid metabolic disorders, and insulin resistance, which in turn exacerbate the development of metabolic syndrome. Targeting this critical node, current therapeutic strategies have evolved from natural compounds and microbial bioactive substances to nanodelivery systems and gene-editing tools, demonstrating potential in inhibiting adipogenesis and improving metabolic abnormalities in both *in vivo* and *in vitro* models. Future research should further elucidate the fine regulatory mechanisms of C/EBPα in adipose cell heterogeneity, develop highly selective small-molecule inhibitors or degraders to reduce off-target effects, and enhance targeting efficiency through tissue-specific nanodelivery technologies. Meanwhile, the exploration of precision regulatory approaches based on single-cell omics and epigenetics, as well as multi-target combination intervention strategies, will open new avenues for personalized treatments for obesity and related metabolic diseases.
